# Supplementary material for: Not-So-Sweet Dreams: Plasma and IgG N-Glycome in the Severe Form of the Obstructive Sleep Apnea
Source: Biomolecules. 2023 May 23;13(6):880. doi: 10.3390/biom13060880 (PMC10296007; doi:10.3390/biom13060880)
Supplement: Supplementary file 1 [file biomolecules-13-00880-s001.zip › biomolecules-2384872-supplementary.pdf]

Supplementary Table S1. The glycan structures corresponding to each of the glycan peaks (GP) are shown. In the case of multiple structures per glycan peak, the upper structure is the major one, and the lower one is minor in abundance/

Peak #	Structure	Composition	Ratio of coeluted structures
GP1	FA2	H3N4F1	
GP2	FA2B	H3N5F1	major structure
	M5	H5N2	low abundance
GP3	A2BG1	H4N5	
GP4	FA2[6]G1	H4N4F1	
GP5	FA2[3]G1	H4N4F1	
GP6	FA2[6]BG1	H4N5F1	
GP7	M6	H6N2	major structure
	FA2[3]BG1	H4N5F1	low abundance
GP8	A2G2	H5N4	
GP9	A2BG2	H5N5	
GP10	FA2G2	H5N4F1	
GP11	FA2BG2	H5N5F	
GP12	M7	H7N2	major structure
	A2G2S1	H5N4S1	major structure
	A1M4G1S1	H5N3S1	low abundance
	A2BG1S1	H4N5S1	low abundance
GP13	FA2G1S1	H4N4F1S1	major structure
	FA2BG1S1	H4N5F1S1	low abundance
GP14	A2G2S1	H5N4S1	
GP15	A2BG2S1	H5N5S1	
GP16	FA2G2S1	H5N4F1S1	
GP17	FA2BG2S1	H5N5F1S1	
GP18	A2G2S2	H5N4S2	major structure
	FA2G2S2	H5N4F1S2	low abundance
GP19	M9	H9N2	
GP20	A2G2S2	H5N4S2	
GP21	A2G2S2	H5N4S2	major structure
	A3G3S1	H6N5S1	low abundance
	FA2G2S2	H5N4F1S2	low abundance
	A2BG2S2	H5N5S2	low abundance
	A3F1G3S1	H6N5F1S1	low abundance
GP22	FA2G2S2	H5N4F1S2	
GP23	FA2BG2S2	H5N5F1S2	
GP24	A3G3S2	H6N5S2	major structure
	A3F1G3S1	H6N5F1S1	low abundance
GP25	A3G3S2	H6N5S2	major structure
	FA2F1G2S2	H5N4F2S2	low abundance

	A3F1G3S2	H6N5F1S2	low abundance
GP26	A3G3S2	H6N5S2	major structure
	FA3G3S2	H6N5F1S2	low abundance
GP27	A3F1G3S2	H6N5F1S2	major structure
	A3G3S3	H6N5S3	low abundance
GP28	A3G3S3	H6N5S3	major structure
	A3F1G3S2	H6N5F1S2	low abundance
GP29	A3G3S3	H6N5S3	major structure
	A3F1G3S2	H6N5F1S2	low abundance
GP30	A3G3S3	H6N5S3	major structure
	A3F1G3S3	H6N5F1S3	low abundance
GP31	FA3G3S3	H6N5F1S3	major structure
	A3G3S3	H6N5S3	low abundance
GP32	A3G3S3	H6N5S3	
GP33	A3F1G3S3	H6N5F1S3	
GP34	FA3G3S3	H6N5F1S3	major structure
	A4G4S3	H7N6S3	low abundance
GP35	FA3F1G3S3	H6N5F2S3	major structure
	A4F1G4S3	H7N6F1S3	low abundance
GP36	A4G4S3	H7N6S3	major structure
	A4F1G4S3	H7N6F1S3	low abundance
GP37	A4G4S4	H7N6S4	major structure
	A4F1G4S3	H7N6F1S3	low abundance
GP38	A4G4S4	H7N6S4	major structure
	A4F1G4S4	H7N6F1S4	low abundance
	A4F1G4S3	H7N6F1S3	low abundance
GP39	A4F1G4S4	H7N6F1S4	major structure
	A4F2G4S4	H7N6F2S4	low abundance

Supplementary Table S2. The glycan structures corresponding to each of the IgG glycan peaks in CGE (P) are shown/

**Peak**	**Glycan structure**	**Composition**
P1	A2G2S2	H5N4S2
P2	A2BG2S2	H5N5S2
P3	FA2G2S2	H5N4F1S2
P4	FA2BG2S2	H5N5F1S2
P5	A2[6]G1S1	H4N4S1
P6	A2[3]G1S1	H4N4S1
P7	FA2[6]G1S1	H4N4F1S1
P8	FA2[3]G1S1	H4N4F1S1
P9	A2G2[6]S1	H5N4S1
P10	A2G2[3]S1	H5N4S1
P11	A2BG2S1	H5N5S1
P12	FA2G2S1, M5, A2	H5N4F1S1

P13	FA2BG2S1	H5N5F1S1
P14	A2B	H3N5
P15	FA2	H3N4F1
P16	A2[6]G1	H4N4
P17	A2[3]G1	H4N4
P18	FA2B	H3N5F1
P19	A2B[6]G1	H4N5
P20	A2B[3]G1	H4N5
P21	FA2[6]G1	H4N4F1
P22	FA2[3]G1	H4N4F1
P23	FA2B[6]G1, A2G2	H4N5F1
P24	FA2B[3]G1	H4N5F1
P25	A2BG2	H5N5
P26	FA2G2	H5N4F1
P27	FA2BG2	H5N5F
